# Long non‐coding RNA HOTAIR/microRNA‐206 sponge regulates STC2 and further influences cell biological functions in head and neck squamous cell carcinoma

**DOI:** 10.1111/cpr.12651

**Published:** 2019-07-11

**Authors:** Tiancheng Li, Yao Qin, Zhen Zhen, Hong Shen, Tiechuan Cong, Erik Schiferle, Shuifang Xiao

**Affiliations:** ^1^ Department of Otorhinolaryngology‐Head and Neck Surgery Peking University First Hospital Beijing China; ^2^ Center for Cancer Research Massachusetts General Hospital and Harvard Medical School Boston Massachusetts

**Keywords:** head and neck squamous cell carcinoma, invasion, long non‐coding RNA homeobox transcript antisense RNA, microRNA‐206, migration, proliferation, stanniocalcin‐2

## Abstract

**Objective:**

It is essential to characterize underlying molecular mechanism associated with head and neck squamous cell carcinoma (HNSCC) and identify promising therapeutic targets. Herein, we explored role of homeobox transcript antisense RNA (HOTAIR) in HNSCC to regulate stanniocalcin‐2 (STC2) by sponging microRNA‐206 (miR‐206).

**Methods:**

HNSCC‐related differentially expressed genes and regulation network amongst HOTAIR, miR‐206 and STC2 were identified. Next, effect of HOTAIR on cell biological functions of HNSCC was identified after transfection of cells with HOTAIR overexpressed plasmids or siRNA against HOTAIR. PI3K/AKT signalling pathway‐related gene expression was measured after miR‐206 and STC2 were suppressed. Cell invasion, migration and proliferation were assessed. Finally, tumour growth was assessed to determine the effects of HOTAIR/miR‐206/STC2 axis in vivo.

**Results:**

HOTAIR specifically bound to miR‐206 and miR‐206 targeted STC2. Downregulated HOTAIR or upregulated miR‐206 suppressed HNSCC cell proliferation, invasion and migration. miR‐206 inhibited PI3K/AKT signalling pathway by down‐regulating STC2. Besides, silenced HOTAIR or overexpressed miR‐206 repressed the tumour growth of nude mice with HNSCC.

**Conclusion:**

HOTAIR regulated HNSCC cell biological functions by binding to miR‐206 through STC2.

## 1 INTRODUCTION

Head and neck squamous cell carcinoma (HNSCC) is the sixth prevalent malignancy worldwide, with rising incidence of 600 000 newly diagnosed every year and a five‐year mortality of 50%.^1^ HNSCC is a heterogeneous entity comprising of tumours arising from the oral cavity, oropharynx, larynx and hypopharynx.^2^ Tobacco use and infection with human papillomavirus as well as alcohol consumption carry a highly increasing risk of HNSCC.^3^ Irrespective of the advancements in the treatments for HNSCC, such as surgery, chemo‐ and radiotherapy, HNSCC prognosis remains unfavourable over the past decade.^4^ HNSCC‐related mortality remains high due to the distant metastases and resistance to chemo‐ and radiotherapy.^5^ Recent evidence suggests that a deeper understanding of molecular mechanisms associated with HNSCC could develop high clinical significance to improve the therapeutic approaches for this disease.^6^

Stanniocalcin‐2 (STC2) involves in calcium and phosphate homoeostasis.^7^ Besides, STC2 expression has been widely determined in metastatic cancers with a vital role played in metastasis and progression of lung cancer.^8^ Interestingly, STC2 has been verified as target of microRNA‐184 (miR‐184), and miR‐184 could suppress STC2 impeding the proliferation, invasiveness and migration capacity of glioblastoma cells.^9^ Evidences have been presented supporting the functions of microRNAs (miRNAs) in regulating the metastatic process of tumours in various cancers, serving as oncogenes or tumour suppressors.^10^ Moreover, the tumour‐suppressing role of miR‐206 has been demonstrated in several malignancies, such as ovarian cancer.^11^ Notably, accumulating evidence has delineated roles of long non‐coding RNAs (lncRNAs) in cancer initiation and progression by regulating miRNAs.^12^ Especially, lncRNA homeobox transcript antisense RNA (HOTAIR) sponges miR‐331‐3p to promote tumorigenesis of gastric cancer.^13^ In addition, lncRNAs, as tumour suppressors or oncogenes, involves in fundamental biological processes like cell proliferation, apoptosis and tumorigenesis.^14^, ^15^ Furthermore, lncRNAs are implicated in the carcinogenesis and progression of HNSCC.^16^ Previous studies have identified upregulated HOTAIR expression in multiple cancer types, such as colorectal cancers and gastric cancer.^17^, ^18^ Meanwhile, a correlation between HOTAIR overexpression and breast cancer metastasis and prognosis as well as hepatocellular carcinoma has been delineated in existing researches.^19^, ^20^ These findings led to a hypothesis that HOTAIR participated in development of HNSCC by serving as a ceRNA of miR‐206.

## 2 MATERIALS AND METHODS

### 2.1 Ethical statement

The study was approved by Institution Review Board of Peking University First Hospital. All animal experiments were conducted with the principles of the National Institute of Animal Health Care Guidelines. All operations were performed after discussion and approved by the Animal Care and Use Committee of Peking University First Hospital.

### 2.2 Cell treatment

The HNSCC cell lines Tu686, TSCCA and Cal27 were purchased from American Type Culture Collection (Manassas, VA, USA). The Hep2 cell line was from Central Laboratory of Peking University First Hospital. The Tb3.1 cell line was from China Xiehe cell bank (Beijing, China). The human immortalized oral epithelial cell (HIOEC) was from Shanghai Cell Bank of the Chinese Academy of Sciences (Shanghai, China). Tb3.1 cell line was cultured in Roswell park memorial institute 1640 (RPMI‐1640) complete medium, and the other cell lines were cultured in minimum Eagle's medium (MEM) complete medium (all with 10% FBS) at 37°C in 5% CO_2_ and finally sub‐cultured. HNSCC cells following HOTAIR treatment HNSCC cells treated with si‐HOTAIR.

### 2.3 Fluorescence in situ hybridization (FISH)

Ribo^TM^ lncRNA FISH probe Mix (Red) and Ribo^TM^ miR‐206 FISH probe Mix (Green) (Guangzhou RIBOBIO Co., Ltd., Guangzhou, Guangdong, China) were performed. Briefly, the HNSCC cells were inoculated into cover slips placed in a 6‐well plate for 1 day, fixed, then treated with proteinase K (2 μg/mL), glycine and acetamidine reagent, and finally added with 250 μL pre‐hybrid solution at 42°C for 1 hour. Cells were added with 250 μL hybrid solution containing the probe (300 ng/mL) at 42°C overnight. After 3 washes with PBSTween (PBST), cells were added with 4’,6‐diamidino‐2‐phenylindole staining solution for nuclear staining and then added into a 24‐well plate and incubated for 5 minutes. After that, cells were sealed with anti‐fluorescent quencher. Five different fields were observed and photographed under a fluorescence microscope (Olympus Optical Co., Ltd., Tokyo, Japan).

### 2.4 Dual luciferase reporter gene assay

The STC2 3'UTR gene fragment was synthesized and introduced to pMIR‐reporter. Mutation (Mut) sites were designed. Target fragment was inserted into pMIR‐reporter reporter plasmid by T4 DNA ligase. Next, pMIR‐STC2‐Wt and pMIR‐STC2‐Mut were transfected with miR‐206 mimic to HEK‐293T cells (CRL‐1415, Shanghai Xin Yu Biotech Co., Ltd., Shanghai, China), respectively. Luciferase assay kit (RG005, Shanghai Beyotime Biotechnology Co., Ltd., Shanghai, China) was used to measure luciferase activity. Relationship between HOTAIR and miR‐206 was also verified.

### 2.5 RNA‐Binding protein immunoprecipitation (RIP)

HNSCC cells were lysed by radioimmunoprecipitation assay for 5 minutes. One proportion of cell extract served as input, and the remaining incubated with antibody and magnetic beads for binding. Magnetic bead‐antibody complex was added with RIP wash buffer and lysed cell at 4°C overnight. Thereafter, magnetic bead‐protein complex was obtained. Sample and input were treated with proteinase K separately to obtain RNA for PCR. argonaute (AGO) (ab32381, 1:50, Abcam Inc, Cambridge, MA, USA). Immunoglobulin (IgG; ab109489, 1:100, Abcam Inc, Cambridge, MA, USA) was served as NC.

### 2.6 RNA isolation and quantitation

Total RNA was obtained. Primers (Table 1) were designed and synthesized by Takara (Takara. Kyoto, Japan). Reverse transcription was conducted as TaqMan MicroRNA Assays Reverse Transcription Primer (4366596, Thermo scientific, Waltham, MA, USA). Quantitative PCR was performed using SYBR^®^ Premix Ex Taq^TM^ II Kit (RR820A, Xingzhi Biotechnology Co., Ltd., Guangzhou, Guangdong, China) in the ABI PRISM^®^ 7300 system (Prism^®^ 7300, Shanghai Kunke Instrument Equipment Co., Ltd., Shanghai, China). U6 served as an internal control for miR‐206 and GAPDH (abs830032, Absin Bioscience Inc, Shanghai, China) for others. The 2^−ΔΔCt ^formula was used.

**Table 1:** Primer sequence for RT‐qPCR

Target genes	Forward primer	Reverse primer
miR‐206	5'‐CAGATCCGATTGGAATGTAAGG‐3'	5'‐TATGCTTGTTCTCGTCTCTGTGTC‐3'
STC2	5'‐GGTGGACAGAACCAAGCTCTC‐3'	5'‐ CGTTTGGGTGGCTCTTGCTA‐3'
HOTAIR	5'‐GGTAGAAAAAGCAACCACGAAGC‐3'	5'‐ACATAAACCTCTGTCTGTGAGTGCC‐3'
Akt	5'‐CCTCCACGACATCGCACTG‐3'	5'‐TCACAAAGAGCCCTCCATTATCA‐3'
PI3K	5'‐CATCACTTCCTCCTGCTCTAT‐3'	5'‐CAGTTGTTGGCAATCTTCTTC‐3'
U6	5’‐CTCGCTTCGGCAGCACA‐3’	5’‐AACGCTTCACGAATTTGCGT‐3’
GAPDH	5'‐ATGGAGAAGGCTGGGGCTC‐3'	5'‐ AAGTTGTCATGGATGACCTTG‐3'

Abbreviations: AKT, mammalian target of rapamycin; GADPH, glyceraldehyde‐3‐phosphate dehydrogenase; HOTAIR, homeobox transcript antisense RNA; miR‐206, microRNA‐206; PI3K, phosphatidylinositol‐3‐kinase; STC2, stanniocalcin‐2; RT‐qPCR, reverse transcription quantitative polymerase chain reaction.

### 2.7 Western blot analysis

Head and neck squamous cell carcinoma tissues were ground into a homogenate and added with lysis buffer to isolate total protein. Next, protein was separated by electrophoresis and transferred onto a nitrocellulose membrane, which was blocked with 5% skim milk at 4°C overnight and incubated overnight with primary rabbit anti‐human polyclonal antibodies to STC2 (1:500, ab63057); AKT (1:500, ab8805); p‐AKT (1:500, ab38449); and PI3K (1:500, ab127617); p‐PI3K (1:500, Y607; ab182651). Subsequently, membrane was added with secondary antibody to horseradish peroxidase‐labelled goat anti‐rabbit IgG (1:100, ab109489, Abcam Inc, Cambridge, MA, USA) at 37°C for 1 hour, and immersed in electro‐chemiluminescence solution for imaging, after which relative level of protein was analysed.

### 2.8 Cell counting kit‐8 (CCK8) assay

Head and neck squamous cell carcinoma cells were added with 5 g/L CCK‐8 (20 μL/well). The culture was terminated after 4 hours of incubation under conditions devoid of light. Then, dimethyl sulphoxide was added under conditions devoid of light. The absorbance (A) value at 490 nm was measured by a microplate reader (Infinite 200, Tecan, Switzerland). Growth curve was drawn with A as ordinate and time (h) as abscissa.

### 2.9 Transwell assay

Cells were seeded in a 6‐well plate for 48 hours, resuspended in serum‐free DMEM, with cell density to 3 × 10^5^ cells/mL. Then, 100 μL cell suspension was added to apical chamber and 500 μL DMEM containing 10% FBS to basolateral chamber in a 37°C incubator with 5% CO_2._ After 24 hours, chamber was added with methanol for 10 minutes and crystal violet for 10 minutes. Cells in apical chamber were sealed with a neutral resin in a coverslip. Six randomly selected fields were observed with a microscope (100×) for counting.

### 2.10 Scratch test

Cells (1 × 10^6^ cells/mL) were added into 6‐well plate till grew on the plate and then starved for 12 hours. Lines were drawn vertically on the plate, and the scratched cells were washed off. Photographs were taken at 0 and 48 hours. Fifteen lines were evenly distributed in scratch photograph. The width of scratches across the lines was measured. Cell migration (%) = (1‐scratch width/initial scratch width) × 100%.

### 2.11 EdU staining

The cells were cultured with 50 μmol/L EdU (EdU labelling kit, Ribobio, Guangzhou, China) for 12 hours, fixed with polyformaldehyde and cultured in 5% glycine for 5 minutes. After being treated with 0.5% Triton X‐100, the cells were added with Triton X‐100, then anti‐EdU antibody and then stained with Hoechst 33342.

### 2.12 Flow cytometry

Cells were fixed by ethanol for 1 hour, and then incubated with RNase (GE101‐01, TransGen Biotech, Beijing, China) at 37°C for 40 minutes, and with PI (Sigma‐Aldrich, Shanghai, China) at 4°C for 2 hours. Fluorescence at 488 nm was measured by a flow cytometer (CytoFLEX, Beckman Coulter, CA 92821, USA).

With Annexin‐V‐FITC cell apoptosis detection kit (APOAF‐20TST, Sigma, USA), 1 × 10^6^ cells were suspended in Annexin‐V‐FITC/PI solution (1:2: 50) for 15 minutes and then added with HEPES buffer. FITC and PI fluorescence were detected at 488 nm.

### 2.13 Xenograft tumour in nude mice

Athymic female nude mice (4‐6 weeks) from Animal Experimental Center of Peking University First Hospital (Beijing, China) were raised at (25 ~ 27°C) and constant humidity (45% ~ 50%). The HNSCC cells (1 × 10^7^ cells/mL) were made into cell suspension and then inoculated into axilla of nude mice. The tumour volume (TV) was calculated as TV = 0.5 × a × b^2^ (a, longest diameter, b, shortest diameter of tumour), and growth curve was drawn. After 5 weeks, mice were euthanized with tumours weighed.

### 2.14 Statistical analysis

Data were processed using SPSS 21.0 statistical software (IBM Corp., Armonk, NY, USA), with normality and homogeneity tested. The data with normal distribution were presented as mean ± standard deviation. The independent sample *t* test was employed for statistical analysis between two groups, and the comparison amongst groups was analysed by one‐way analysis of variance (ANOVA). The pairwise comparison amongst multiple groups was performed by Tukey's post hoc test. Besides, the TV at different time points was compared by repeated measures ANOVA. All the cell experiments were conducted in triplicates. *P* < 0.05 was statistically significant.

## 3 RESULTS

### 3.1 High HOTAIR expression in HNSCC cells

**Figure 1:**
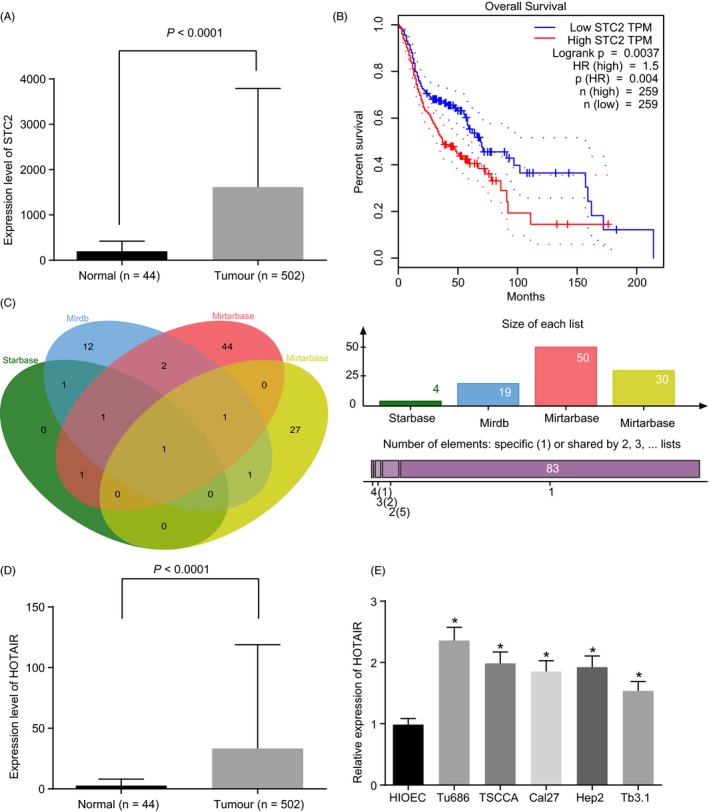
HOTAIR is upregulated in HNSCC cells. A, the expression of STC2 in HNSCC tissues and normal tissues in TCGA database; B, the survival of patients with different expression of STC2; C, the intersection of miRNA targeting STC2 predicted by four prediction websites; D, the expression of HOTAIR in HNSCC tissues and normal tissues; E, the expression of HOTAIR in five HNSCC cell lines and normal oral epithelial cell line detected by RT‐qPCR; One‐way ANOVA was used for statistical analysis, and the experiment was performed in triplicates; data were represented as mean ± standard deviation; *P < 0.05 vs HIOEC cell; STC2, stanniocalcin‐2; HNSCC, head and neck squamous cell carcinoma; RT‐qPCR, reverse transcription quantitative polymerase chain reaction; HOTAIR, homeobox transcript antisense RNA; ANOVA, analysis of variance

DEGs related to HNSCC from TCGA database revealed that STC2 was highly expressed in HNSCC and correlated to HNSCC prognosis (Figure 1A‐B). Various online sources verified that miR‐206 targeted STC2 (Figure 1C) (miRDB, Starbase website, miRSearch website and the  Mirtarbase website). HOTAIR was highly expressed in HNSCC (Figure 1D). RT‐qPCR demonstrated that HOTAIR expression in HNSCC cells was higher than that in HIOEC cells (*P* < 0.05). HOTAIR was differentially expressed in 5 HNSCC cell lines (Figure 1E). In this experiment, Tu686 cells with the highest HOTAIR expression were selected.

### 3.2 HOTAIR silencing inhibits HNSCC cell biological functions

**Figure 2:**
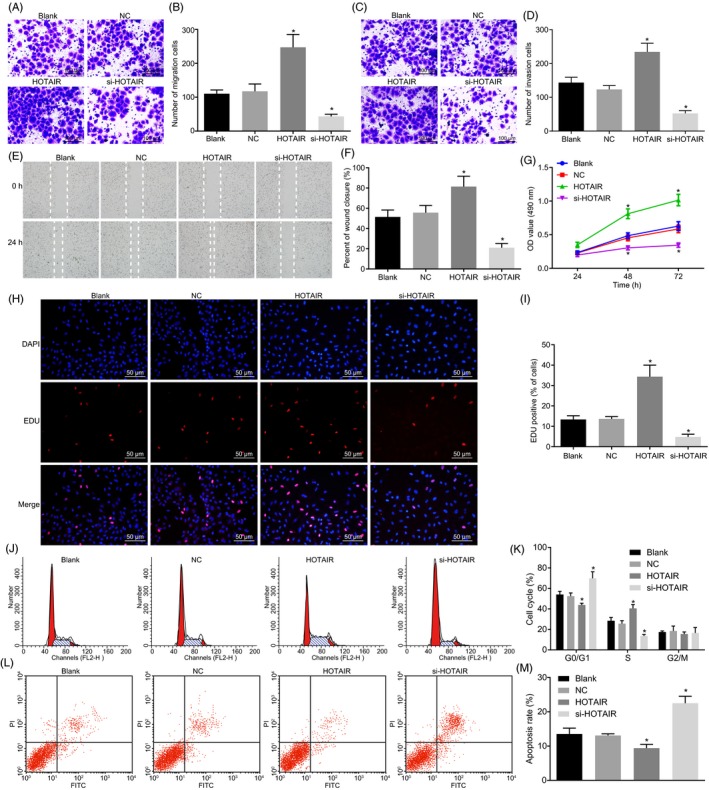
HOTAIR silencing suppresses the proliferation, migration and invasion while elevating apoptosis of HNSCC cells. A‐D, the migration and invasion of HNSCC cells treated with HOTAIR overexpressed plasmids or siRNA against HOTAIR detected by transwell assay (×100); E‐F, the cell migration and scratch healing rate HNSCC cells by scratch test; G, cell growth curve detected by CCK‐8; H‐I, proliferation and fluorescence intensity assayed by EdU; J‐K, cell cycle and cell cycle ratio detected by flow cytometry; L‐M, cell apoptosis assayed by flow cytometry. One‐way ANOVA was used for statistical analysis amongst groups, and the experiment was performed in triplicates; data were represented as mean ± standard deviation; *P < 0.05 vs the blank or NC group

Transwell results showed that compared with blank and NC, HNSCC cells following HOTAIR treatment presented increased number of migrating cells and invasive cells (all *P* < 0.05), while HNSCC cells treated with si‐HOTAIR exhibited a decreased number of migrating cells and invasive cells (*P* < 0.05). Besides, scratch test also verified that HNSCC cells following HOTAIR treatment exhibited accelerated healing of scratches and higher healing rate at 48 hours. While the results were reciprocal in HNSCC cells treated with si‐HOTAIR when compared with blank and NC (*P* < 0.05) (Figure 2A‐F).

Furthermore, CCK‐8 assay and EdU assay revealed that compared with blank and NC, HNSCC cells following HOTAIR treatment showed an elevated cell growth rate and increased fluorescence intensity (all *P* < 0.05), while HNSCC cells treated with si‐HOTAIR presented the opposite trends (*P* < 0.05) (Figure 2G‐I).

Flow cytometry showed that in comparison to blank and NC, proportion of cells in G0/G1 phase decreased, but that in S phase cells increased, and apoptotic rate decreased in HNSCC cells following HOTAIR treatment. On the contrary, the results in si‐HOTAIR group were the opposite (Figure 2J‐M).

### 3.3 Subcellular localization of HOTAIR

**Figure 3:**
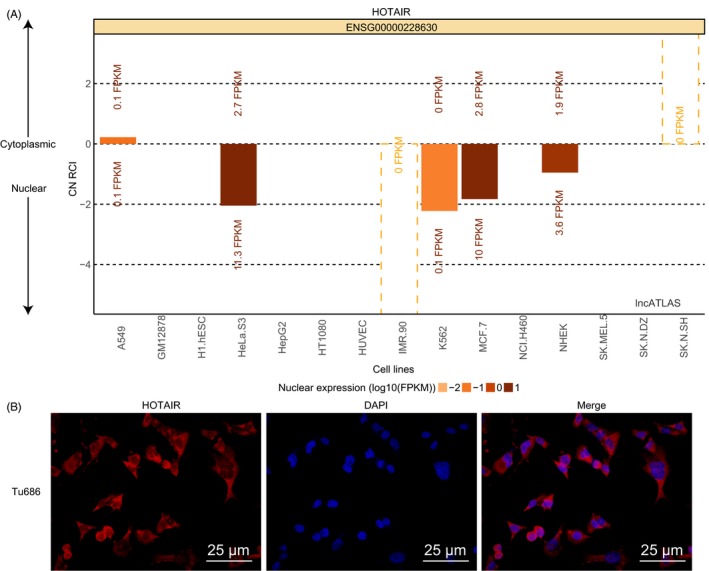
HOTAIR is expressed in both the nucleus and cytoplasm of Tu686 cells. A, subcellular localization of HOTAIR predicted by bioinformatics website; B, subcellular localization of HOTAIR detected by FISH; FISH, fluorescence in situ hybridization

Through the analysis on http://lncatlas.crg.eu/, HOTAIR was expressed in both nucleus and cytoplasm (Figure 3A). Furthermore, FISH assay showed that in the Tu686 cells, HOTAIR was expressed in both the nucleus and cytoplasm. The green part depicted HOTAIR expression, and the blue part represented the nucleus (Figure 3B).

### 3.4 HOTAIR competitively binds to miR‐206

**Figure 4:**
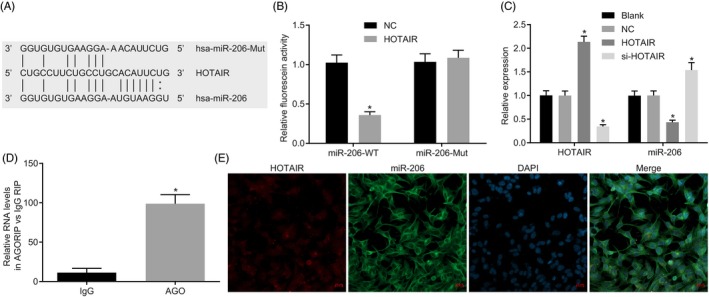
HOTAIR sponges miR‐206. A, the binding site between HOTAIR, miR‐206 and miR‐206‐Mut predicted by bioinformatics website; B, the luciferase activity in each group detected by dual luciferase reporter gene assay; C, the expression of miR‐206 in each group measured by RT‐qPCR; D, the binding of HOTAIR and AGO assessed by RIP; E, HOTAIR and miR‐206 localization by fluorescence double staining. The measurement data amongst multiple groups in panel B and C were analysed by one‐way ANOVA; the independent sample *t* test was used for statistical analysis between two groups; the data in FIGURE D were analysed by independent sample *t* test; the experiment was performed in triplicates. *P < 0.05 vs the miR‐206‐Mut, blank or IgG groups. RIP, RNA‐binding protein immunoprecipitation; IgG, immunoglobulin G

The binding of HOTAIR and miR‐206 was predicted through bioinformatics website RNA22 (Figure 4A). Dual luciferase reporter assay (Figure 4B) revealed that in contrast to NC, luciferase activity in the miR‐206‐Wt had decreased (*P* < 0.05), but that of miR‐206‐Mut almost remained the same (*P* > 0.05). RT‐qPCR (Figure 4C) revealed that in HNSCC cells following HOTAIR treatment HOTAIR expression was increased, miR‐206 expression was decreased, while HNSCC cells treated with si‐HOTAIR showed opposite results, suggesting that HOTAIR competitively bound to miR‐206 and inhibited its expression. RIP (Figure 4D) showed that HOTAIR could bind to AGO protein. Dual luciferase reporter assay showed that HOTAIR could co‐localize with miR‐206 in cytoplasm (Figure 4E).

### 3.5 miR‐206 directly targets STC2

**Figure 5:**
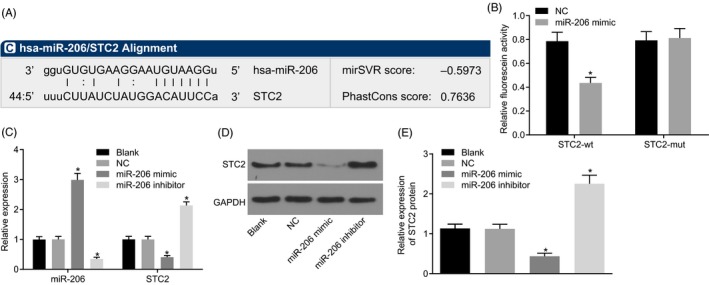
miR‐206 targets STC2. A, verification of the targeting relationship between miR‐206 and STC2; B, the luciferase activity of STC2‐Wt and STC2‐Mut detected by dual luciferase reporter assay; C, the expression of STC2 and miR‐206 assessed by RT‐qPCR; D, the grey value of STC2 protein band in response to the treatment of miR‐206 mimic or miR‐206 inhibitor; E, the protein level of STC2 determined by Western blot analysis; one‐way ANOVA was used for different factors analysis, and the independent sample *t* test was used for statistical analysis between two groups; the experiment was performed in triplicates; data were represented as mean ± standard deviation; *, *P* < 0.05 vs the NC group or the blank group. Wt; wide type; Mut, mutant

The analysis from bioinformatics website microRNA.org revealed the presence of a specific binding region between 3'UTR of STC2 and miR‐206 sequences (Figure 5A). Dual luciferase reporter assay (Figure 5B) demonstrated that miR‐206 inhibited luciferase activity of STC2‐Wt only (*P* < 0.05) (*P* > 0.05). RT‐qPCR and Western blot analysis (Figure 5C‐E) suggested that miR‐206 mimic group exhibited lower STC2 expression and higher miR‐206 expression than the blank and NC (all *P* < 0.05). An increased STC2 expression was observed, with reduced miR‐206 expression in the miR‐206 inhibitor group (*P* < 0.05).

### 3.6 miR‐206 targets STC2 to inhibit PI3K/Akt signalling pathway activation

**Figure 6:**
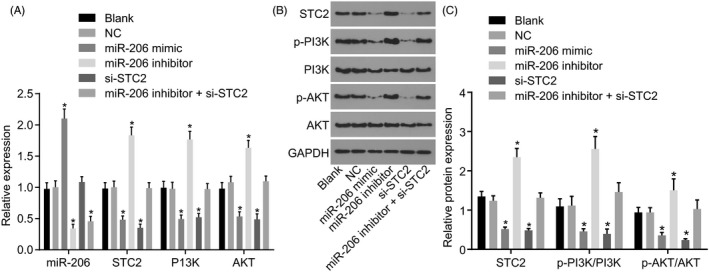
miR‐206 inhibits the activation of the PI3K/AKT signalling pathway by targeting STC2. A, the expression of miR‐206 and mRNA levels of STC2, PI3K and AKT detected by RT‐qPCR; B, the grey value of STC2, PI3K, p‐PI3K, AKT and p‐AKT; C, the protein levels of STC2, p‐PI3K/PI3K and p‐AKT/AKT determined by Western blot analysis; one‐way ANOVA was used for analysis amongst groups, and the experiment was performed in triplicates; data were represented as mean ± standard deviation; *P < 0.05 vs the blank or the NC group

In contrast to blank and NC, miR‐206 mimic increased miR‐206 expression (*P* < 0.05), along with decreased STC2 expression, p‐PI3K/PI3K and p‐AKT/AKT (*P* < 0.05); si‐STC2 reduced levels of STC2, p‐PI3K/PI3K and p‐AKT/AKT (*P* < 0.05); miR‐206 expression was reduced while the STC2 expression, p‐PI3K/PI3K and p‐AKT/AKT were elevated following miR‐206 inhibitor treatment (*P* < 0.05); miR‐206 inhibitor + si‐STC2 treatment reduced miR‐206 expression with no changes in STC2 expression, p‐PI3K/PI3K and p‐AKT/AKT (all *P* > 0.05) (Figure 6). Overexpression of miR‐206 or silencing STC2 inhibited PI3K/AKT signalling pathway activation.

### 3.7 Overexpressed miR‐206 or silenced STC2 suppresses HNSCC cell biological function via PI3K/AKT signalling pathway

**Figure 7:**
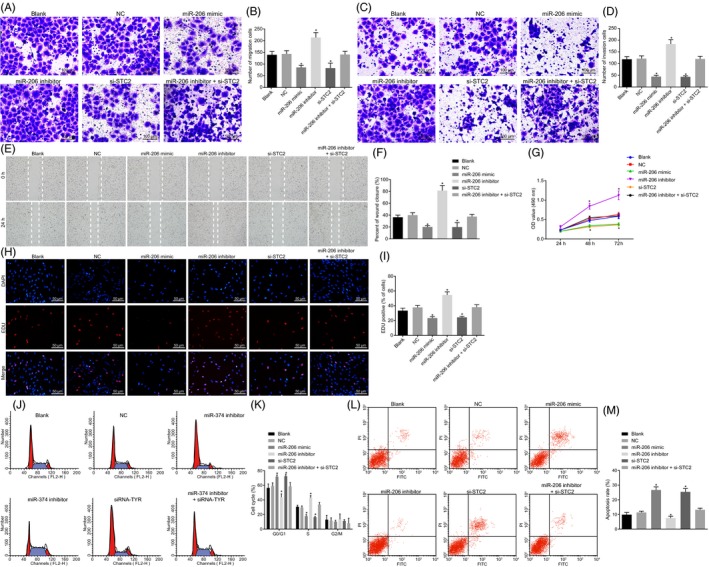
miR‐206 overexpression or STC2 silencing leads to the inhibition of HNSCC cell proliferation, migration and invasion, and elevation of apoptosis. A‐B, HNSCC cell migration detected by transwell assay (× 100); C‐D, HNSCC cell invasion by transwell assay; E‐F, HNSCC cell migration ability and healing rate by scratch test; G, CCK‐8 for detection of growth of HNSCC cells; H, EDU assay for proliferation of HNSCC cells; I, EDU fluorescence intensity statistical results in each group; J‐K, flow cytometry for cell cycle entry and cell cycle ratio of HNSCC cells; L‐M, flow cytometry for HNSCC cell apoptosis in each group. One‐way ANOVA was used for analysis amongst groups, and the experiment was performed in triplicates; data were represented as mean ± standard deviation; *P < 0.05 vs the blank or NC group

To further assess the effect of miR‐206/STC2/PI3K/Akt signalling pathway on the biological function of HNSCC cells, transwell assay was firstly used to detect HNSCC cell migration (Figure 7A‐B) and invasion (Figure 7C‐D). The results proved that in contrast to the blank and NC, miR‐206 inhibitor increased number of migrating cells and invasive cells, while miR‐206 mimic and si‐STC2 exhibited opposite trends (*P* < 0.05). Moreover, scratch test also verified that miR‐206 inhibitor accelerated healing of scratches, while co‐treatment of miR‐206 inhibitor + si‐STC2 revealed opposite trends. Thus, effects of miR‐206 inhibitor were reversed by si‐STC2 (Figure 7E‐F).

CCK‐8 and EdU assays were employed to further analyse the changes in the proliferation of HNSCC cells after transfection. HNSCC cells treatment with miR‐206 inhibitor exhibited a higher cell growth rate and higher fluorescence intensity than blank and NC (*P* < 0.05), while that treated with miR‐206 mimic or si‐STC2 presented opposite results (*P* < 0.05); co‐treatment of miR‐206 inhibitor + si‐STC2 showed reduced cell growth rate and fluorescence intensity in contrast to miR‐206 inhibitor (Figure 7G‐I) (*P < *0.05).

Furthermore, we detected the cell cycle entry and apoptotic rate by flow cytometry. In HNSCC cells after miR‐206 mimic or si‐STC2 treatment, the G0/G1 phase cell cycle entry increased, S phase proportion decreased and apoptotic rate increased, while it was opposite in HNSCC cells after miR‐206 inhibitor + si‐STC2 treatment. After cells treated with miR‐206 inhibitor + si‐STC2, si‐STC2 reversed role of miR‐206 inhibitor (Figure 7J‐M). miR‐206 affected the cell cycle and apoptosis of HNSCC by regulating STC2 (Figure 7J‐M).

### 3.8 STC2 silencing reverses the biological function changes of Tu686 cells induced by HOTAIR

**Figure 8:**
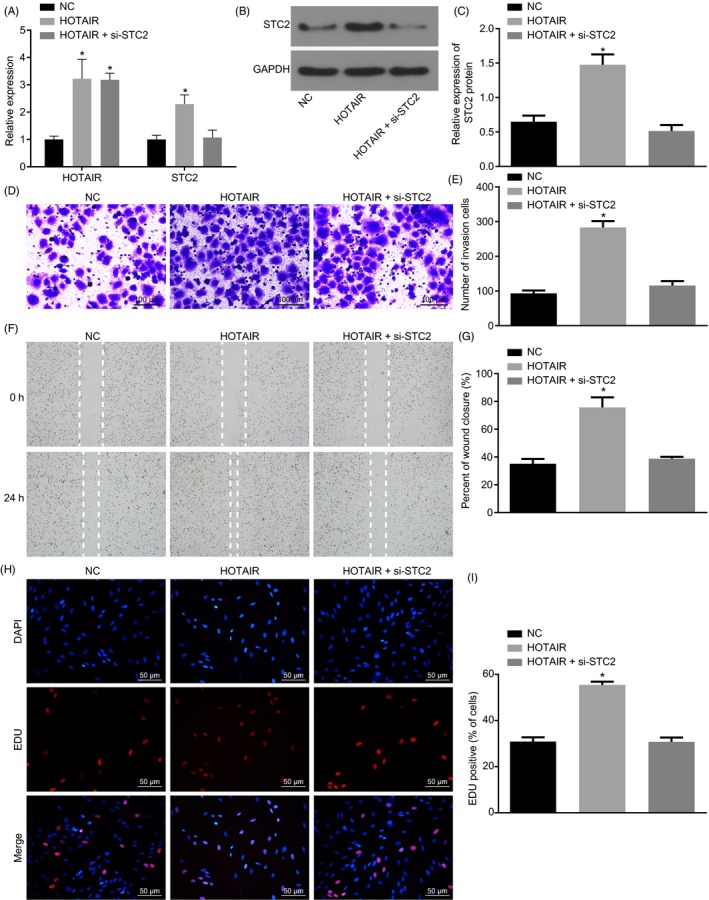
STC2 reverses the biological function changes of Tu686 cells induced by HOTAIR. A, expression of HOTAIR and STC2 in each group by RT‐qPCR; B‐C, STC2 protein in each group by Western blot analysis; D‐E, invasion of HNSCC cells by transwell assay (×100); F‐G, migration ability and scratch healing rate of HNSCC cells by scratch test; H‐I, EDU detection of cell proliferation and fluorescence intensity in each group. One‐way ANOVA was used for analysis amongst groups, and the experiment was performed in triplicates; data were represented as mean ± standard deviation; *P < 0.05 vs the NC group

From the previous results, HOTAIR binding to miR‐206 regulates STC2 expression. To determine whether STC2 can reverse the changes of cell biological function induced by HOTAIR, HNSCC cells were treated with NC, HOTAIR or HOTAIR + si‐STC2. Based on RT‐qPCR and Western blot analysis, compared with HNSCC cells following NC treatment, HOTAIR and STC2 levels in HNSCC cells following HOTAIR treatment were increased, while HOTAIR level was increased, but STC2 levels remained statistically similar in HNSCC cells treated with HOTAIR + si‐STC2 (Figure 8A‐C).

Further, invasion and proliferation of cells were tested by transwell assay and scratch test. The results showed that compared with HNSCC cells were treated with NC, invasion and proliferation of cells in HNSCC cells following HOTAIR treatment increased. When treated with HOTAIR + si‐STC2, the invasion and proliferation of cells induced by HOTAIR were reversed by si‐STC2 (Figure 8D‐I). Therefore, STC2 silencing could reverse the biological function changes of Tu686 cells induced by HOTAIR.

### 3.9 HOTAIR silencing or miR‐206 upregulation reduces tumour growth in nude mice

**Figure 9:**
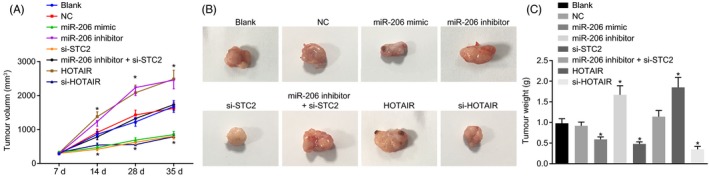
HOTAIR silencing or miR‐206 overexpression suppresses tumour growth in nude mice injected with transfected HNSCC cells. A, the tumour volume of nude mice measured by a vernier caliper; B, xenograft tumours in nude mice observed by naked eyes; C, tumour weight of nude mice; one‐way ANOVA was used for analysis amongst groups, and the experiment was performed in triplicates; data were represented as mean ± standard deviation; *P < 0.05 vs the blank or NC group

The results (Figure 9A‐C) suggested that tumour volume in nude mice increased with time. Mice treated with miR‐206 mimic, si‐STC2 or si‐HOTAIR showed smaller tumour volume and lower weight (all *P* < 0.05) while mice following miR‐206 inhibitor or HOTAIR treatment presented contrary trends. These results showed that HOTAIR silencing or miR‐206 overexpression inhibited tumour growth in vivo.

## 4 DISCUSSION

HNSCC is characterized by high recurrence, metastasis and unsatisfactory treatment results.^21^ There is evidence indicating that the dysregulation of lncRNAs functions crucially in the genesis and development of HNSCC.^22^ For instance, HOTAIR exerts a prognostic effect over biological functions of HNSCC.^23^ Notably, HOTAIR has been demonstrated to be correlated with cell apoptosis and proliferation in various human malignancies, including HNSCC.^24^ Thus, we investigated the role of HOTAIR in HNSCC, and findings revealed that silencing HOTAIR upregulate miR‐206 to downregulate STC2, thus inhibiting HNSCC biological functions.

HOTAIR was highly expressed in HNSCC cells, and silenced HOTAIR consequently inhibited HNSCC cell proliferation, invasion and migration. LncRNAs are involved in many cellular processes like cell proliferation, migration and invasion.^25^ HOTAIR expression is also enhanced in non‐small‐cell lung cancer (NSCLC) and knockdown of HOTAIR suppresses NSCLC cell invasion and metastasis.^26^ Additionally, overexpression of HOTAIR can be referred to as a biomarker in gastric cancer (GC) and silenced HOTAIR could inhibit the cell invasion and viability in GC.^13^

Besides, we found that HOTAIR could competitively bind to miR‐206 as a ceRNA. LncRNAs could negatively regulate miRNAs by serving as ceRNAs of miRNAs.^27^ In addition, there are evidences showing that HOTAIR could bind to miR‐217 in renal cell carcinoma.^28^ Moreover, existing research has shown that HOTAIR regulates Rab22a expression in ovarian cancer through competitively sponging miR‐37.^29^

Additionally, miR‐206 targeted STC2 to suppress HNSCC cell proliferation, migration and invasion through the PI3K/AKT signalling pathway. miR‐206 reduces biological functions of lung squamous cell carcinoma (SCC), thereby repressing cell proliferation, migration and invasion in lung SCC.^30^ Furthermore, previous research observed overexpressed miR‐206 to inhibit cell proliferation and migration in colorectal cancer, highlighting the tumour‐suppressive abilities of miR‐206.^31^ Most importantly, consistent with our results, Liu *et al* observed miR‐206 was downregulated in HNSCC, and overexpressed miR‐206 could inhibit cell growth, migration and invasion in HNSCC.^32^ Notably, STC2 upregulation increased HNSCC cell proliferation, invasion and migration, tumour growth, and metastasis, revealing that STC2 could be a novel strategy for HNSCC treatment.^33^ Further, STC2 is a target of miR‐206, and miR‐206 could downregulate STC2 expression. In consistency with our results, miR‐206 inhibited tumour growth and metastasis in GC via targeting STC2.^34^ The impact of activated PI3K/AKT signalling pathway is significant in various fundamental biological activities.^35^ Likewise, the PI3K/AKT signalling pathway regulated cell biological functions in HNSCC.^36^ Furthermore, overexpressed miR‐206 suppressed lung cancer cell migration and invasion via inhibition of the PI3K/AKT/mTOR signalling pathway.^37^

**Figure 10:**
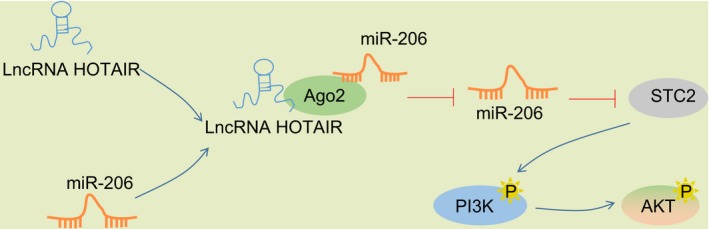
HOTAIR competitively binds to miR‐206, thereby promoting STC2 expression, activating PI3K/AKT signalling pathway

In conclusion, silencing HOTAIR could inhibit HNSCC biological functions *via* STC2 downregulation by competitively binding to miR‐206. HOTAIR could competitively bind to miR‐206, thereby stimulating STC2 expression, activating PI3K/AKT signalling pathway (Figure 10). Thus, HOTAIR silencing can serve as a therapeutic target for HNSCC. However, further studies with larger sample sizes are needed to elucidate specific mechanisms of HOTAIR in HNSCC.

## CONFLICT OF INTEREST

None.

## AUTHOR'S CONTRIBUTION

TCL and YQ designed the study. ZZ collated the data. Erik Schiferle and SFX carried out data analyses and produced the initial draft of the manuscript. HS and TCC contributed to drafting and polishing the manuscript. All authors have read and approved the final submitted manuscript.

## References

[1] Zou AE , Zheng H , Saad MA , et al. The non‐coding landscape of head and neck squamous cell carcinoma. Oncotarget. 2016;7:51211‐51222. 27323410 10.18632/oncotarget.9979 PMC5239470

[2] Economopoulou P , Perisanidis C , Giotakis EI , Psyrri A . The emerging role of immunotherapy in head and neck squamous cell carcinoma (HNSCC): anti‐tumor immunity and clinical applications. Ann Transl Med. 2016;4:173. 27275486 10.21037/atm.2016.03.34 PMC4876265

[3] Stransky N , Egloff AM , Tward AD , et al. The mutational landscape of head and neck squamous cell carcinoma. Science. 2011;333:1157‐1160. 21798893 10.1126/science.1208130 PMC3415217

[4] Victoria Martinez B , Dhahbi JM , Nunez Lopez YO , et al. Circulating small non‐coding RNA signature in head and neck squamous cell carcinoma. Oncotarget. 2015;6:19246‐19263. 26057471 10.18632/oncotarget.4266 PMC4662488

[5] Chen C , Wei Y , Hummel M , et al. Evidence for epithelial‐mesenchymal transition in cancer stem cells of head and neck squamous cell carcinoma. PLoS ONE. 2011;6:e16466. 21304586 10.1371/journal.pone.0016466 PMC3029362

[6] Wu D , Cheng J , Sun G , et al. p70S6K promotes IL‐6‐induced epithelial‐mesenchymal transition and metastasis of head and neck squamous cell carcinoma. Oncotarget. 2016;7:36539‐36550. 27174914 10.18632/oncotarget.9282 PMC5095019

[7] Kita Y , Mimori K , Iwatsuki M , et al. STC2: a predictive marker for lymph node metastasis in esophageal squamous‐cell carcinoma. Ann Surg Oncol. 2011;18:261‐272. 20734150 10.1245/s10434-010-1271-1

[8] Na S‐S , Aldonza MB , Sung H‐J , et al. Stanniocalcin‐2 (STC2): A potential lung cancer biomarker promotes lung cancer metastasis and progression. Biochim Biophys Acta. 2015;1854:668‐676. 25463045 10.1016/j.bbapap.2014.11.002

[9] Feng L , Ma J , Ji H , Liu Y , Hu W . MiR‐184 retarded the proliferation, invasiveness and migration of glioblastoma cells by repressing stanniocalcin‐2. Pathol Oncol Res. 2018;24:853‐860. 28887636 10.1007/s12253-017-0298-z

[10] Marini F , Luzi E , Brandi ML . MicroRNA role in thyroid cancer development. J Thyroid Res. 2011;2011:407123. 21687652 10.4061/2011/407123 PMC3112511

[11] Li S , Li Y , Wen Z , et al. microRNA‐206 overexpression inhibits cellular proliferation and invasion of estrogen receptor alpha‐positive ovarian cancer cells. Mol Med Rep. 2014;9:1703‐1708. 24604205 10.3892/mmr.2014.2021

[12] Zhou M , Wang X , Shi H , et al. Characterization of long non‐coding RNA‐associated ceRNA network to reveal potential prognostic lncRNA biomarkers in human ovarian cancer. Oncotarget. 2016;7:12598‐12611. 26863568 10.18632/oncotarget.7181 PMC4914307

[13] Liu X‐H , Sun M , Nie F‐Q , et al. Lnc RNA HOTAIR functions as a competing endogenous RNA to regulate HER2 expression by sponging miR‐331‐3p in gastric cancer. Mol Cancer. 2014;13:92. 24775712 10.1186/1476-4598-13-92 PMC4021402

[14] Liu Q , Huang J , Zhou N , et al. LncRNA loc285194 is a p53‐regulated tumor suppressor. Nucleic Acids Res. 2013;41:4976‐4987. 23558749 10.1093/nar/gkt182 PMC3643595

[15] Sun M , Nie F , Wang Y , et al. LncRNA HOXA11‐AS promotes proliferation and invasion of gastric cancer by scaffolding the chromatin modification factors PRC2, LSD1, and DNMT1. Cancer Res. 2016;76:6299‐6310. 27651312 10.1158/0008-5472.CAN-16-0356

[16] Zhang Z‐L , Zhao L‐J , Chai L , et al. Seven LncRNA‐mRNA based risk score predicts the survival of head and neck squamous cell carcinoma. Sci Rep. 2017;7:309. 28331188 10.1038/s41598-017-00252-2 PMC5428014

[17] Guan G‐F , Zhang D‐J , Wen L‐J , et al. Overexpression of lncRNA H19/miR‐675 promotes tumorigenesis in head and neck squamous cell carcinoma. Int J Med Sci. 2016;13:914‐922. 27994496 10.7150/ijms.16571 PMC5165684

[18] Endo H , Shiroki T , Nakagawa T , et al. Enhanced expression of long non‐coding RNA HOTAIR is associated with the development of gastric cancer. PLoS ONE. 2013;8:e77070. 24130837 10.1371/journal.pone.0077070 PMC3795022

[19] Sørensen KP , Thomassen M , Tan Q , et al. Long non‐coding RNA HOTAIR is an independent prognostic marker of metastasis in estrogen receptor‐positive primary breast cancer. Breast Cancer Res Treat. 2013;142:529‐536. 24258260 10.1007/s10549-013-2776-7

[20] Yang Z , Zhou L , Wu L‐M , et al. Overexpression of long non‐coding RNA HOTAIR predicts tumor recurrence in hepatocellular carcinoma patients following liver transplantation. Ann Surg Oncol. 2011;18:1243‐1250. 21327457 10.1245/s10434-011-1581-y

[21] Sannigrahi MK , Sharma R , Panda NK , Khullar M . Role of non‐coding RNAs in head and neck squamous cell carcinoma: A narrative review. Oral Dis. 2018;24:1417‐1427. 28941018 10.1111/odi.12782

[22] Haque S‐U , Niu L , Kuhnell D , et al. Differential expression and prognostic value of long non‐coding RNA in HPV‐negative head and neck squamous cell carcinoma. Head Neck. 2018;40:1555‐1564. 29575229 10.1002/hed.25136 PMC6037541

[23] Zou AE , Ku J , Honda TK , et al. Transcriptome sequencing uncovers novel long noncoding and small nucleolar RNAs dysregulated in head and neck squamous cell carcinoma. RNA. 2015;21:1122‐1134. 25904139 10.1261/rna.049262.114 PMC4436665

[24] Kong L , Zhou X , Wu Y , et al. Targeting HOTAIR Induces Mitochondria Related Apoptosis and Inhibits Tumor Growth in Head and Neck Squamous Cell Carcinoma in vitro and in vivo. Curr Mol Med. 2015;15:952‐960. 26592246 10.2174/1566524016666151123112716

[25] Murugan AK , Munirajan AK , Alzahrani AS . Long noncoding RNAs: emerging players in thyroid cancer pathogenesis. Endocr Relat Cancer. 2018;25:R59‐R82. 29146581 10.1530/ERC-17-0188

[26] Liu X‐H , Liu Z‐L , Sun M , Liu J , Wang Z‐X , De W . The long non‐coding RNA HOTAIR indicates a poor prognosis and promotes metastasis in non‐small cell lung cancer. BMC Cancer. 2013;13:464. 24103700 10.1186/1471-2407-13-464 PMC3851855

[27] Chen D‐L , Lu Y‐X , Zhang J‐X , et al. Long non‐coding RNA UICLM promotes colorectal cancer liver metastasis by acting as a ceRNA for microRNA‐215 to regulate ZEB2 expression. Theranostics. 2017;7:4836‐4849. 29187907 10.7150/thno.20942 PMC5706103

[28] Hong Q , Li O , Zheng W , et al. LncRNA HOTAIR regulates HIF‐1alpha/AXL signaling through inhibition of miR‐217 in renal cell carcinoma. Cell Death Dis. 2017;8:e2772. 28492542 10.1038/cddis.2017.181 PMC5520706

[29] Zhang Z , Cheng J , Wu YI , Qiu J , Sun YI , Tong X . LncRNA HOTAIR controls the expression of Rab22a by sponging miR‐373 in ovarian cancer. Mol Med Rep. 2016;14:2465‐2472. 27484896 10.3892/mmr.2016.5572 PMC4991663

[30] Mataki H , Seki N , Chiyomaru T , et al. Tumor‐suppressive microRNA‐206 as a dual inhibitor of MET and EGFR oncogenic signaling in lung squamous cell carcinoma. Int J Oncol. 2015;46:1039‐1050. 25522678 10.3892/ijo.2014.2802

[31] Wang X‐W , Xi X‐Q , Wu J , Wan Y‐Y , Hui H‐X , Cao X‐F . MicroRNA‐206 attenuates tumor proliferation and migration involving the downregulation of NOTCH3 in colorectal cancer. Oncol Rep. 2015;33:1402‐1410. 25607234 10.3892/or.2015.3731

[32] Liu F , Zhao X , Qian Y , Zhang J , Zhang Y , Yin R . MiR‐206 inhibits Head and neck squamous cell carcinoma cell progression by targeting HDAC6 via PTEN/AKT/mTOR pathway. Biomed Pharmacother. 2017;96:229‐237. 28987947 10.1016/j.biopha.2017.08.145

[33] Yang S , Ji Q , Chang B , et al. STC2 promotes head and neck squamous cell carcinoma metastasis through modulating the PI3K/AKT/Snail signaling. Oncotarget. 2017;8:5976‐5991. 27863406 10.18632/oncotarget.13355 PMC5351606

[34] Ren J , Huang H‐J , Gong YU , Yue S , Tang L‐M , Cheng SY . MicroRNA‐206 suppresses gastric cancer cell growth and metastasis. Cell Biosci. 2014;4:26. 24855559 10.1186/2045-3701-4-26 PMC4030529

[35] Cheng L , Luo S , Jin C , Ma H , Zhou H , Jia L . FUT family mediates the multidrug resistance of human hepatocellular carcinoma via the PI3K/Akt signaling pathway. Cell Death Dis. 2013;4:e923. 24232099 10.1038/cddis.2013.450 PMC3847326

[36] Chen Y , Zhao R , Zhao Q , Shao Y , Zhang S . Knockdown of HPIP inhibits the proliferation and invasion of head‐and‐neck squamous cell carcinoma cells by regulating PI3K/Akt signaling pathway. Oncol Res. 2016;24:153‐160. 27458096 10.3727/096504016X14612603423476 PMC7838746

[37] Chen Q‐Y , Jiao D‐M , Wu Y‐Q , et al. MiR‐206 inhibits HGF‐induced epithelial‐mesenchymal transition and angiogenesis in non‐small cell lung cancer via c‐Met /PI3k/Akt/mTOR pathway. Oncotarget. 2016;7:18247‐18261. 26919096 10.18632/oncotarget.7570 PMC4951285

